# The Development of Endoscopic Techniques for Treatment of Walled-Off Pancreatic Necrosis: A Single-Center Experience

**DOI:** 10.1155/2018/8149410

**Published:** 2018-04-01

**Authors:** Mateusz Jagielski, Marian Smoczyński, Anna Jabłońska, Krystian Adrych

**Affiliations:** Department of Gastroenterology and Hepatology, Medical University of Gdańsk, Gdańsk, Poland

## Abstract

**Background:**

Endotherapy is a common method of treatment in patients with symptomatic walled-off pancreatic necrosis (WOPN). The aim of this study is to indicate the potential therapeutic possibilities created by the combination of several new endoscopic techniques and the evaluation of their efficacy in the treatment of WOPN.

**Methods:**

The retrospective analysis of results and complications in the group of 101 patients, who underwent endoscopic treatment of symptomatic WOPN between years 2011 and 2015.

**Results:**

Endoscopic treatment was started in 101 patients (71 men, 30 women; mean age 50.97 years) with symptomatic WOPN. Single transluminal gateway technique (SGT) was used in 93/101 (92.08%) patients. SGT in combination with multiple transluminal gateway technique (MTGT) was exploited in 4/93 (4.30%) patients, while in combination with single transluminal gateway transcystic multiple drainage (SGTMD) in 22/93 (23.66%) patients. Transpapillary access was used in 11/101 (10.89%) patients. 20/101 (19.80%) patients underwent percutaneous drainage. Fluoroscopy-guided endoscopic necrosectomy was performed in 19/101 (18.81%) patients. The combinations of endoscopic techniques depended on the extent of necrosis. Procedure-related complications occurred in 16/101 (15.84%) patients. The mortality rate was 0.99% (1/101 patient). Therapeutic success was achieved in 99/101 (98.02%) patients. The long-term success of endoscopic treatment was achieved in 97/101 (96.04%) patients with symptomatic WOPN.

**Conclusions:**

Application of new endoscopic techniques in the treatment of the patients with symptomatic WOPN significantly improves the efficiency of endotherapy with an acceptable amount of complications.

## 1. Introduction

Walled-off pancreatic necrosis (WOPN) is diagnosed in 15% of patients with severe acute pancreatitis [1]. The most common form of necrotizing pancreatitis is mixed necrosis (parenchymal and peripancreatic) which affects 75%–80% of patients [2]. The less common is peripheral (peripancreatic 20%) and central (pancreatic 5%) types of necrosis [2]. Better results of the conservative treatment of acute pancreatitis allow to delay interventional therapy until the resolution of organ failure, demarcation and liquefaction of necrosis, and hence the formation of WOPN that usually takes place at least four weeks after the onset of symptoms [3–5]. Conservative treatment in the early phase of acute necrotizing pancreatitis and the delay of necrosectomy to the late stage of the disease significantly reduces mortality in this group of patients [6]. Interventional treatment is necessary for patients with clinical symptoms (including infection of necrosis) resulting from the presence of necrotic collection [1, 3].

Transmural endoscopic drainage is a common method of treatment for patients with symptomatic WOPN [1, 3, 7]. Single transluminal gateway technique (SGT) is based on the complete removal of necrotic tissues through a single fistula created between the cavity of necrotic collection and the lumen of the gastrointestinal tract (stomach or duodenum) [7, 8]. The described technique is particularly efficient in the case of unilocular necrotic collections. WOPN in most of the patients takes the form of a multilocular space divided by septa.

Decompression of necrotic collections during interventional treatment results in the formation of separated necrotic areas that are in fact separate cavities (undrained areas) communicating with one another via narrow canals [9]. In such cases, a single access to necrotic collection is insufficient, and as additional access to necrosis is necessary [1, 3], a percutaneous drainage is usually performed [10, 11]. The use of other endoscopic techniques, like the creation of another transmural tract, endoscopic necrosectomy, or active transpapillary drainage, is also possible [7, 9, 12–14]. One method of treatment of WOPN—transpapillary drainage—is rarely described in the current literature and is still considered to be controversial [14].

The combination of several minimally invasive methods of treatment, allowing multiple access to necrotic collection, is an optimal strategy for the treatment of WOPN [1, 3, 10, 11]. Widening of the access to necrotic areas provides better draining conditions and increases the efficiency of treatment [1, 3, 7]. The method of access to WOPN should depend not only on the location of necrosis but also on the experience of the medical center.

Endoscopic drainage of walled-off pancreatic necrosis has been performed in our center since 2001 [7, 15]. Conventional transmural drainage (CTD) was performed between 2001 and 2011 [7]. Since 2011, the fistulas between the lumen of the gastrointestinal tract and the cavity of necrotic collection have been performed with endoscopic ultrasonography (EUS) guidance (EUS-guided drainage) [15]. The evolution and development of endoscopic therapy of WOPN in our medical center have led us to perform this retrospective analysis of efficiency and safety of the methods used.

This paper presents our own experience in the endoscopic treatment of walled-off pancreatic necrosis and indicates the potential therapeutic possibilities created by the combination of several new endoscopic techniques and evaluation of their efficacy in the treatment of WOPN.

## 2. Methods

The study was approved by the Ethics Committee of our Medical University. All patients gave their informed consent for endoscopic procedures.

### 2.1. Qualification to Study

The qualification to endoscopic treatment was based on the clinical picture and the results of imaging studies, mostly contrast-enhanced computed tomography (CECT). The walled-off pancreatic necrosis was diagnosed on the basis of the revised Atlanta classification from 2012 [4, 5]. The presence of necrotic tissues in an EUS image and morphology of an aspirate from the collection (dark brown color and fragments of necrotic tissues) confirmed the diagnosis of WOPN.

### 2.2. Exclusion Criteria

Excluded from the study were patients with WOPN who had no symptoms connected with the presence of pancreatic fluid collection (11 patients). We also excluded patients with symptomatic WOPN in whom EUS showed that the WOPN wall was located more than 15 mm from the gastrointestinal tract wall and endoscopic retrograde pancreatography (ERP) revealed no communication between the main pancreatic duct and the collection cavity (14 patients). Moreover, patients who were qualified to endoscopic drainage on the basis of a clinical picture and CECT image in whom EUS revealed no necrotic tissues (“solid debris”) and morphology of aspirate (clear, serous) suggested pancreatic pseudocyst were also excluded (8 patients).

### 2.3. Study Group

The study group comprised 101 patients with symptomatic walled-off pancreatic necrosis who underwent endoscopic therapy in our department over a 5-year period from 2011 to 2015.

### 2.4. Choice of Endoscopic Treatment Technique

In all patients, an attempt of transmural drainage was made. In the case of patients with symptomatic WOPN, transmural drainage was not performed if the distance between the collection wall and the gastrointestinal wall exceeded 15 mm in EUS. Among patients who did not undergo transmural drainage, those in whom ERP revealed the leak of contrast medium into the necrotic collection were qualified to transpapillary drainage. Furthermore, when transmural drainage did not lead to complete regression of WOPN, in some patients with communication of the main pancreatic duct with collection cavity observed during ERP additional transpapillary, drainage was used. Endoscopic necrosectomy under fluoroscopic guidance was performed when the following criteria were fulfilled: lack of clinical effect or infection of necrotic collection despite the active drainage as well as a large number of necrotic tissues in a fluoroscopic and endosonographic image.

During the early period of the study (years 2011–2013) in the case of ineffective endoscopic drainage and spreading of necrosis outside the lesser omental sac, additional percutaneous drainage was performed. During the later phase of the study (years 2013–2015), if drainage through a single transmural access (single transluminal gateway technique (SGT)) was ineffective, another transmural tract was created (multiple transluminal gateway technique (MTGT)) when there was no communication between the necrotic collection subcavities or multiple access through a single transmural fistula (single transluminal gateway transcystic multiple drainage (SGTMD)) was used, which involved obtaining an additional access to extensive necrotic areas through a single transmural tract.

### 2.5. Procedures

Endoscopic procedures were performed with the use of duodenoscope Pentax ED3490TK and echoendoscope Pentax EG3870UTK. The procedure was performed with deep sedation. In all patients, endoscopic drainage was performed by one endoscopist. All patients received antibiotics before the procedure (ciprofloxacin or ceftriaxone with metronidazole). Routinely, antibiotic treatment was continued for two weeks. An aspirate from the collection was sent for amylase activity and microbial culture, and appropriate culture-directed modification of antibiotics was made.

### 2.6. Transmural Drainage (Single Transluminal Gateway Technique (SGT))

In all patients, an attempt to perform transmural drainage was made. The place of the transmural tract was chosen with EUS guidance. Gastrostomy (or duodenostomy) was created with the use of Giovannini cystostome (Cystotome CST-10, Wilson-Cook). The fistula formed between the gastrointestinal lumen and necrotic cavity was sequentially dilated with an 8 mm or 20 mm balloon dilator (Boston Scientific). A 7Fr or 8Fr nasocystic drain (Balton or Wilson-Cook) and double-pigtail stents 7Fr or 8.5Fr (ZSO-10-5, Wilson-Cook or Mar Flow) were deployed within the necrotic cavity through the transmural tract.

### 2.7. Endoscopic Retrograde Pancreatography (ERP)

**Figure 1:**
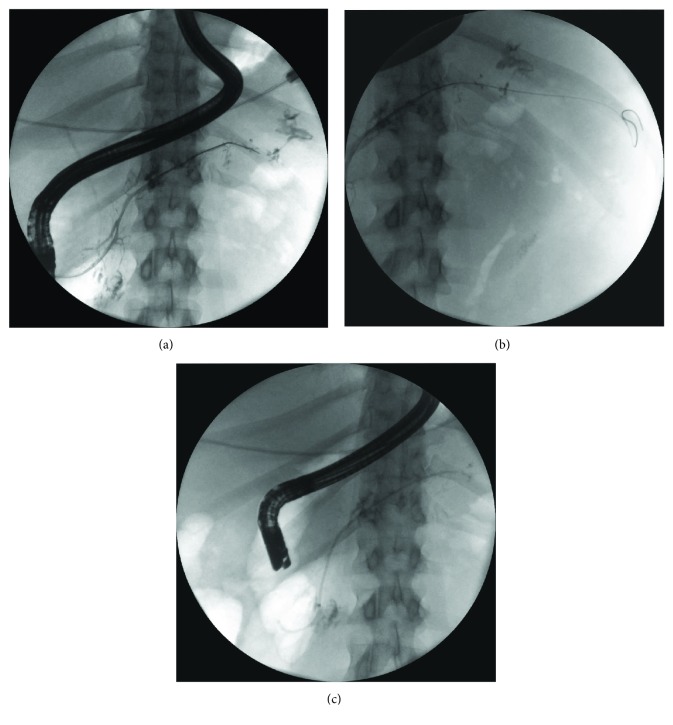
Endoscopic retrograde pancreatography done in the patient with WOPN. Partial disruptions of the main pancreatic duct in the body of pancreas are well visible as well as the complete disruption of MPD in the pancreatic tail (a). The guidewire inserted through the complete disruption of main pancreatic is located in the lumen of WOPN (b). Transpapillary 5Fr 12 cm pancreatic stent was used to bridge the disruption of the pancreatic duct (c).

When the main pancreatic duct leak was observed, sphincterotomy was performed with an Olympus FlowCut KD-301Q0725 sphincterotome and pancreatic stent was placed (passive transpapillary drainage) to bridge the leak—5Fr, 7Fr, 8.5Fr, or 10Fr (Geenen, Zimmon Pancreatic Stent, Wilson-Cook Medical Inc. or Mar Flow) (Figures 1(a)–1(c)). The stent was then replaced with a new one after 6, 12, and 24 months or until the pancreatic duct leak was no longer demonstrated.

### 2.8. Active Transpapillary Drainage

In patients with active transpapillary drainage after sphincterotomy was performed during ERP, the main pancreatic duct was mechanically dilated with bougie dilator 7Fr, 8.5Fr, or 10Fr (Wilson-Cook). Sequentially, a nasocystic drain (7Fr or 8Fr, Balton or Wilson-Cook) and pancreatic stent (5–10Fr, Geenen, Zimmon Pancreatic Stent, Wilson-Cook Medical Inc. or Mar Flow) were placed through the duodenal papilla. The distal tip of nasocystic drain was deployed within the necrotic cavity.

### 2.9. Multiple Transluminal Gateway Technique (MTGT)

In patients qualified to the creation of another transmural tract between the necrotic cavity and the gastrointestinal lumen, the site of fistulotomy was also chosen with EUS guidance. Enterostomy was performed with Giovannini cystostome (Cystotome CST-10, Wilson-Cook). The fistula was dilated with an 8 mm or 20 mm balloon dilator (Boston Scientific). A 7Fr or 8Fr nasocystic drain (Balton or Wilson-Cook) and double-pigtail stents 7Fr or 8.5Fr (ZSO-10-5, Wilson-Cook or Mar Flow) were placed in the necrotic cavity through the transmural tract.

### 2.10. Single Transluminal Gateway Transcystic Multiple Drainage (SGTMD)

**Figure 2:**
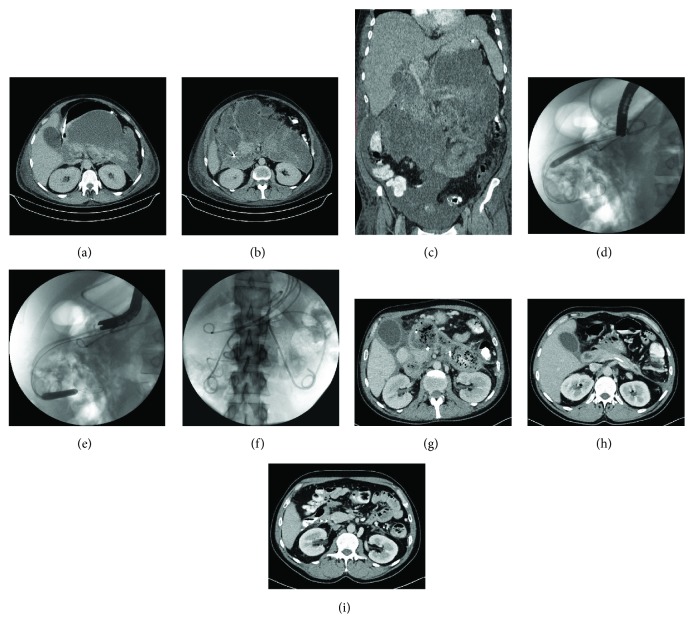
The patient with an extensive walled-off pancreatic necrosis visible in contrast-enhanced computed tomography (a, b, c). SGTMD technique was exploited for treatment (d, e, f, g). CECT performed after the end of endoscopic treatment stated complete regression of WOPN (h, i).

In patients qualified to SGTMD (Figures 2(a)–2(i)), subsequent endoscopic procedures were performed and a guidewire was introduced in the subcavities with fluoroscopy guidance through the transmural tract created between the necrotic collection and the gastrointestinal lumen. The canals between necrotic subcavities were dilated with an 8 mm balloon dilator (Boston Scientific) under fluoroscopy guidance (Figures 2(d) and 2(e)). Afterwards, another 7Fr or 8Fr nasocystic drain (Balton or Wilson-Cook) or double-pigtail stents 7Fr or 8.5Fr (ZSO-10-5, Wilson-Cook or Mar Flow) were introduced through those canals and their distal ends were deployed within necrotic subcavities (Figures 2(f) and 2(g)).

### 2.11. Fluoroscopy-Guided Endoscopic Necrosectomy

**Figure 3:**
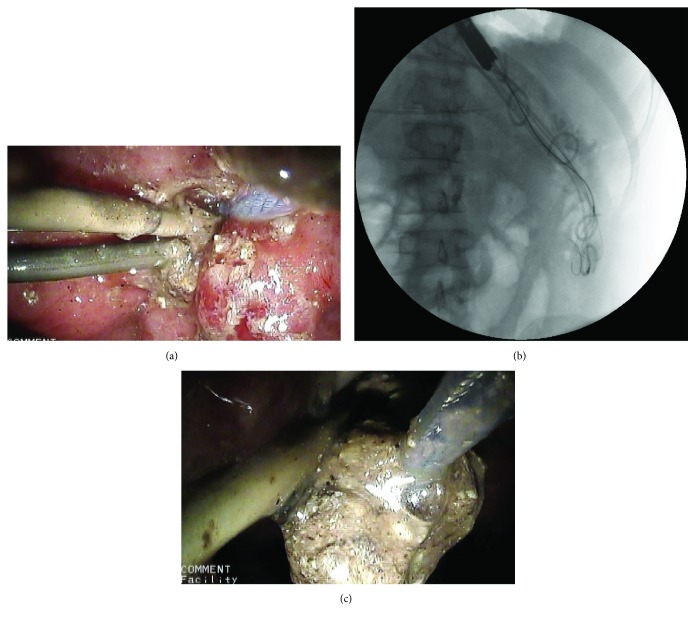
Endoscopic necrosectomy under fluoroscopic guidance. The Dormia basket is positioned in the lumen of necrotic collection (a, b). Numerous fragments of necrotic tissues were thereafter removed from the necrotic cavity during endoscopic necrosectomy (c).

At the beginning of endoscopic necrosectomy procedure, a nasocystic drain was removed. Subsequently, a Dormia basket (FG-V422PR, Olympus) was introduced through the fistula in the necrotic area adjacent to the previously placed transmural stent. Necrotic tissues were removed with the Dormia basket through the transmural tract with fluoroscopy guidance (Figures 3(a)–3(c)). This action was repeated many times during each procedure of necrosectomy. At the end of procedure, another nasocystic drain was deployed.

### 2.12. Drainage System

The nasocystic drains were flushed with saline solution (60–200 mL) every 2 hours within the first 48 hours and then every 4 hours. When there was a clinical suspicion of WOPN infection, the use of antibiotics was prolonged and another microbial culture with antibiogram of necrotic collection contents was performed.

### 2.13. Assessment of Therapeutic Effect

The effect of drainage was monitored every 7 days, mainly with the use of conventional ultrasonography. Contrast-enhanced CT was performed to confirm complete regression of the collection. Active drainage/debridement was finished after the resolution of clinical symptoms and complete regression of the collection or the decrease of the collection's diameter to less than 40 mm.

### 2.14. Definition of Therapeutic Success and Recurrence of the Collection

Therapeutic success was defined as the lack of symptoms and complete regression of the collection or the dimension of the collection less than 40 mm during a three-month follow-up since the end of active drainage. Recurrence of the collection was determined as the collection size > 40 mm or relapse of symptoms during a follow-up. Long-term success was defined as the lack of symptoms and complete regression of the collection or the dimension of the collection less than 40 mm during a follow-up.

### 2.15. Statistical Analysis

All the statistical calculations were performed with the use of data analysis software system StatSoft Inc. (2011) STATISTICA version 10.0 (licensed for the Medical University of Gdansk). Quantitative variables were characterized by arithmetic means, standard deviation, and minimal and maximal values (range), whereas qualitative data were presented by the means of numbers and percentage.

## 3. Results

### 3.1. Patients' Characteristics

Patients' characteristics are presented in Table 1.

Endoscopic treatment was started in 101 patients with symptomatic WOPN. The etiology of acute pancreatitis was alcoholic in 61 patients and nonalcoholic in 40 (23: gallstones, 6: iatrogenic, 2: hypertriglyceridemia, and 9: idiopathic). Two of 101 patients (1.98%) did not complete endotherapy. One patient was referred to surgical treatment because of gastrointestinal perforation during endotherapy. During the operation, perforation was repaired and surgical drainage of WOPN was performed. Another patient died during endoscopic treatment because of splenic artery pseudoaneurysm hemorrhage.

### 3.2. Infection of Walled-Off Pancreatic Necrosis

The WOPN infection was diagnosed on the basis of positive microbial culture in 31/101 (30.69%) patients. The most common pathogens cultured in the necrotic contents were *Escherichia coli*, *Enterococcus faecalis*, and *Staphylococcus epidermidis.* In 12/101 (11.88%) patients, sepsis with positive blood culture was observed during endotherapy.

### 3.3. Access Route to Necrotic Collection

**Figure 4:**
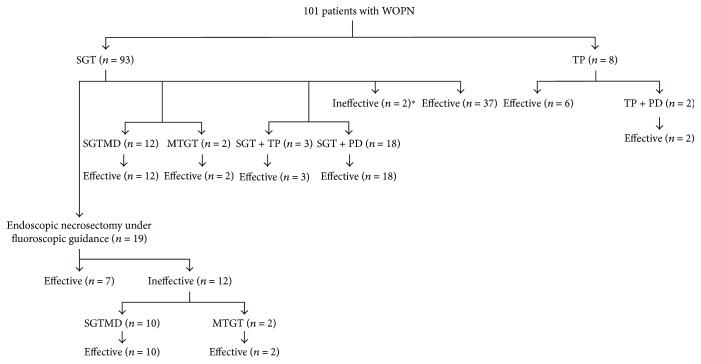
The access routes to necrotic collection and the results of treatment with the use of endoscopic techniques in patients with WOPN. PD: percutaneous drainage; TP: active transpapillary drainage; SGT: single transluminal gateway techniques; MTGT: multiple transluminal gateway techniques; SGTMD: single transluminal gateway transcystic multiple drainage; Effective: therapeutic success; Ineffective: without therapeutic success. ^∗^Two did not complete endotherapy: one patient was referred to surgical treatment, and another one patient died during endoscopic treatment.

The access routes to necrotic collection and endoscopic management techniques applied to our patients are presented in Figure 4.

Transmural access was used in 93/101 (92.08%) patients (gastric: 86, duodenal: 7). In 8 patients, the transmural route was not performed, because the distance between the gastrointestinal lumen and the necrotic cavity exceeded 15 mm. Transpapillary access was used in 11 patients. Twenty patients underwent percutaneous drainage.

### 3.4. Endoscopic Retrograde Pancreatography (ERP)

ERP was performed in 89 patients in order to diagnose and treat a main pancreatic duct leak. In 79/89 (88.76%) patients, the main pancreatic duct leak required transpapillary pancreatic stent placement. A partial rupture of the main pancreatic duct was diagnosed in 51/79 (64.56%) patients, and a complete rupture in 28/79 (35.44%) patients.

### 3.5. Duration and Effectiveness of Therapy

Therapeutic success was achieved in 99/101 (98.02%) patients. The mean duration period of active drainage of WOPN was 23 (4–173) days. The mean number of procedures was 4.35 (1–27).

### 3.6. Complications of Treatment

Procedure-related complications occurred in 16/101 (15.84%) patients. One patient required surgical treatment of endotherapy complications. The most common complication—gastrointestinal bleeding—was observed in 9/101 (8.91%) patients. Because of gastrointestinal bleeding, seven patients were required packed red blood cell transfusions, one underwent endovascular embolization of the hepatoduodenal artery pseudoaneurysm, and one patient died because of splenic artery pseudoaneurysm hemorrhage. Transmural stent migration into the WOPN cavity was stated in 3/101 (2.97%) patients. In all cases, the stent was retrieved endoscopically with the Dormia basket. Gastrointestinal perforation was diagnosed in 2/101 (1.98%) patients. One of them required surgical management, and the other one was treated conservatively.

### 3.7. Mortality

The mortality rate was 0.99% (1/101 patient). The cause of death was splenic artery pseudoaneurysm bleeding.

### 3.8. Long-Term Success

The mean follow-up period was 32 (15–74) months. During the follow-up, the recurrence of WOPN was observed in 9/101 (8.91%) patients. In two patients, the recurrence was managed surgically and in seven endoscopically. The long-term success of endoscopic treatment was achieved in 97/101 (96.04%) patients with symptomatic WOPN.

## 4. Discussion

For the last two decades, we have observed the development of endoscopic management of WOPN. Baron et al. were the first who presented the results of treatment with the use of single transmural access in patients with WOPN [8]. Therapeutic success was achieved in 9 of 11 patients (81.82%) [8]. In our study, the SGT was efficient in 37 of 93 patients (39.79%). The use of another endoscopic technique or additional route of access to the necrotic collection was necessary in 54 remaining patients (54/93 (58.06%)).

The first reports concerning transmural drainage of pancreatic necrosis presented the creation of fistulas between the gastrointestinal lumen and the necrotic collection cavity that were measuring 10–12 mm in diameter [8]. With the development of this method, the diameter of the fistula was enlarged even up to 20 mm, which allowed the insertion of fiberscope into the area of necrosis and the performance of endoscopic necrosectomy [13]. The implementation of endoscopic necrosectomy is considered to be the next step that improved the efficiency of endotherapy. Seifert et al. proved the beneficial effect of endoscopic necrosectomy in 75 of 93 (81%) patients with WOPN [16]. Complications were observed in 24 of 93 (26%) of patients, and the mortality rate was 7.5% [16]. In a multicenter study, Gardner et al. reached the therapeutic success in 95 of 104 (91%) patients who underwent endoscopic necrosectomy [17]. Comparable results of treatment (with the efficiency of 86%) were described by Rische et al. [18]. Gardner et al. reported the complication rate of 14% and the mortality rate of 5.8% [17]. By comparison, in the study conducted by Rische et al., the complications occurred in 13% of patients and no lethal complications were observed [18]. All patients from the above studies underwent endoscopic necrosectomy. In our work, the indications for endoscopic necrosectomy under fluoroscopic guidance during transmural drainage were stated in 19 (18.81%) patients. A good therapeutic effect was achieved in all of the 19 patients; however, some of them required another access to necrotic areas with the use of other minimally invasive methods of treatment. The complications of endoscopic necrosectomy occurred in 4 of 19 (21.05%) patients. No lethal complications were observed.

Serious complications of air embolism were encountered in some studies that were connected with the inflation of gas into the necrotic collection to enable visualization of its cavity [16, 17, 19]. Seifert et al. reported two cases of air embolism (2.08%) in patients who underwent endoscopic necrosectomy (one case of fatal pulmonary embolism and one patient with cerebral infarction after air embolism via a persistent foramen ovale) [16]. Application of carbon dioxide [20] instead of room air [16, 17, 19] for endoscopic insufflations during endoscopic necrosectomy reduces the risk of the occurrence of air embolism [20]. In our study, we presented an endoscopic necrosectomy technique that is based on the removal of necrotic tissues under fluoroscopic guidance without the need to introduce fiberscope into the necrotic collection cavity and without its inflation with gas [21], which also eliminates the risk of air embolism. Furthermore, our method of necrosectomy [21] is less traumatic compared to the one described by many authors [16–20].

The reports describing the use of transpapillary drainage as the only way of access to pancreatic necrosis are rare in the literature [14]. Transpapillary drainage is much more often combined with the transmural or percutaneous route as multiplied access to the necrotic cavity [3, 7]. In our study, a transpapillary drainage was performed in eight patients with WOPN, in whom there was no chance to create a transmural fistula and the necrotic collection was communicating with the main pancreatic duct. Transpapillary drainage as the only route of access to necrosis was efficient in 6 of 8 (75%) patients. Two of 8 (25%) patients required additional percutaneous access.

The use of percutaneous access during the endoscopic treatment of WOPN decreases the number of both endoscopic and radiological procedures, shortens the duration of hospital stay, and increases the efficiency of treatment in patients with pancreatic necrosis [10, 11]. During the early stage of our study in the case of ineffective transmural drainage (lack of complete regression of WOPN), a transpapillary (3/93 patients (3.23%)) or percutaneous (18/93 patients (19.35%)) drainage was performed as another access route, which improved drainage conditions and increased the effectiveness of therapy. Therefore, percutaneous drainage can be used as both single and additional routes of access to necrotic collection in the management of WOPN. In a systematic review of eleven studies, including 384 patients, percutaneous drainage appeared to be effective as the only method of treatment in more than half of patients (55.7%) [22]. In our study, percutaneous drainage was not used as the only access to WOPN.

With the development of endoscopic techniques of treatment, both the diameter and the number of fistulas were increased. In 2011, Varadarajulu et al. have first described multiple transluminal gateway technique based on the creation of multiple transmural tracts between the gastrointestinal lumen and the WOPN cavity [12]. The authors proved that the use of several (2-3) routes of access to pancreatic necrosis (MTGT) is a more effective method of treatment than single transluminal gateway technique [12]. Varadarajulu et al. achieved therapeutic success in 11/12 (91.7%) patients who underwent MTGT in comparison to 25/48 (52.1%) patients treated with SGT [12]. Three years later, Mukai et al. presented the technique that enables access to extensive necrotic areas through a single fistula (SGTMD) without the need to create an additional transmural route [9]. Mukai et al. observed a good therapeutic effect in all of 5 patients (100%). In the same report, the authors showed therapeutic success in all of 9 patients (100%) with WOPN that were treated with the use of MTGT [9].

The presented evolution of endoscopic techniques has significantly increased the efficiency of transmural drainage. In our center, the development of endoscopic methods allowed us to replace percutaneous drainage in patients with ineffective single transmural drainage with MTGT in 4/93 (4.3%) patients or SGTMD in 22/93 (23.66%) patients depending on the extent of necrosis. The use of these techniques in combination with endoscopic necrosectomy improved the effectiveness of WOPN treatment up to 100%.

In 2015, Mukai et al. published a report that showed a good therapeutic effect of various endoscopic techniques in 86/89 (96.6%) patients with WOPN and their complication rate was 12.4% (11/89 patients) [23]. In our study, the treatment success was achieved in 99/101 (98.02%) patients with a complication rate of 15.84% (16/101).

Endoscopic treatment is an alternative for other minimally invasive techniques for the treatment of WOPN, especially for percutaneous drainage. Presented results of endoscopic treatment of patients with WOPN provoke the discussion about the therapeutic strategy in this group of patients. On the basis of our own experience shown in this publication, it seems that the use of percutaneous drainage and other minimally invasive techniques with percutaneous access as the first stage of treatment could be effectively replaced with endoscopic transmural drainage. In a large group of patients with WOPN, endotherapy can remain the only method of treatment. Our study proved that SGT was an effective method of management in half (50.54%) of patients with symptomatic WOPN. In the case of extensive necrotic areas adjacent to the gastrointestinal tract, the creation of additional transmural access is efficient (MTGT). When the excessive distance between the gastrointestinal wall and the necrotic collection hinders the formation of another transmural tract, a good therapeutic effect can be achieved by additional drainage of extensive necrosis through a single fistula (SGTMD). In a selected group of patients, endoscopic necrosectomy combined with active transmural drainage improves the results of treatment. Transpapillary drainage remains an effective method of treatment in patients without transmural access to necrotic collection that is communicating with the main pancreatic duct.

The choice of an access route to WOPN should depend on the extent of necrosis and the experience of a medical center [1, 3, 19]. Comparing the results of treatment presented in this report with the publication originating from our center concerning 112 patients with WOPN who underwent conventional drainage [7], we conclude that with the increase of experience in the field of drainage procedures there is an improvement in the efficiency and safety of therapy.

The main limitations of our study are lack of randomization, retrospective character, relatively short follow-up, and highly selected group of patients from a single center. Although our report presents the experience of one center, we consider the fact that all endoscopic procedures were conducted by one endoscopist to be its advantage, which allows a reliable comparison of the endoscopic treatment results over the years. Recently, there have been many publications confirming the efficiency of self-expandable metal stents (SEMS) in the transmural drainage of pancreatic fluid collections [24, 25]. In our study, plastic transmural stents were used for all patients. The use of SEMS in our work could have reduced the duration of treatment and the number of endoscopic procedures.

Summing up, our report presents the evolution of various endoscopic techniques and their use in the management of WOPN. In our center, the development of transmural endoscopic methods of treatment has significantly reduced the use of other minimally invasive techniques, particularly percutaneous drainage. We have shown that the endoscopic treatment of patients with WOPN is an effective method with an acceptable number of complications.

The application of new endoscopic techniques in the treatment of the patients with symptomatic WOPN significantly improves the efficiency of endotherapy with an acceptable amount of complications. The development of endoscopic methods of treatment for WOPN has significantly reduced the use of other minimally invasive techniques.

## Figures and Tables

**Table 1:** Characteristics of the patients with WOPN who underwent endoscopic treatment.

	All patients (*n* = 101)
Age, mean (range)	50.97 (21–85)
Sex, *n*, men (%)	71 (70.3%)
Etiology, *n* (%)	
Alcoholic	61 (60.4%)
Nonalcoholic	40 (39.6%)
WOPN size (cm), mean (range)	12.4 (5.0–36.3)
WOPN type, *n* (%)	
Pancreatic parenchymal necrosis alone	23 (22.77%)
Peripancreatic necrosis alone	10 (9.90%)
Both pancreatic and peripancreatic necroses	68 (67.33%)
Time from the acute bout of pancreatitis (weeks), mean (range)	19.17 (3–80)
Main indication to start endotherapy, *n* (%)	
Infected necrosis	31 (30.69%)
Abdominal pain	96 (95.05%)
Gastrointestinal obstruction	40 (39.60%)
Jaundice	8 (7.92%)
Weight loss	38 (37.62%)
